# Pediatric Stroke Presenting as a Seizure

**DOI:** 10.1155/2014/838537

**Published:** 2014-12-22

**Authors:** Katie L. Ahmadzadeh, Vartika Bhardwaj, Steven A. Johnson, Kathleen E. Kane

**Affiliations:** Department of Emergency Medicine, Lehigh Valley Hospital, USF Morsani COM, CC & I-78, Allentown, PA 18103, USA

## Abstract

*Background.* Childhood arterial ischemic stroke (AIS) is rare and may be difficult to diagnose. Management of acute stroke in any age group is time sensitive, so awareness of the manifestations and appropriate diagnostic procedures for pediatric AIS is vital to establishing care. We present a pediatric case of arterial ischemic stroke that presented to the emergency department (ED) after two seizures. *Case Report.* A five-year-old female with an existing seizure disorder presented to a pediatric ED after having two seizures. Postictal upon arrival, she underwent a computed tomography (CT) scan of her head. Family reported that she had complained of a severe headache and vomited; her seizures were described as different from those she had experienced in the past. Loss of grey white matter differentiation on the CT warranted magnetic resonance imaging (MRI), which demonstrated a right-sided stroke. After a complicated course in the hospital, the patient was discharged to a rehabilitation hospital. *Why Should an Emergency Physician Be Aware of This?* It is important that emergency physicians recognize that a seizure may be the initial symptom of a pediatric stroke regardless of an established seizure history. Pediatric seizures are relatively common; however consideration of the diagnosis of pediatric stroke may prevent unnecessary delays in treatment.

## 1. Introduction

Childhood arterial ischemic stroke (AIS) is a subset of pediatric stroke that encompasses patients aged from 29 days to 18 years [1]. For the clinician, the recognition of AIS in children is a challenge because it is infrequent and the presentation is varied and is nonspecific [2]. It is reported to have a mortality rate of 2–11% with persistent neurological defect in 68–73% of surviving patients [1]. Prompt diagnosis and treatment of pediatric AIS is critical to improve outcomes for patients [1, 2]. AIS, estimated to affect 1–13/100,000 children per year in North America and Europe, occurs when flow-limiting arterial stenosis or thromboembolic occlusion causes irreversible tissue injury [3–5]. Forty percent of stroke cases in children are reported to occur under one year of age [6]. Recurrence is common, estimated at 7–20% within 5 years for all children with AIS [1]. Etiologies include cerebral arteriopathies, abnormalities in vasculature, infection, trauma to the head or neck, congenital heart disease, sickle cell anemia, genetic/metabolic disease, and prothrombotic abnormalities [1]. For the emergency physician, diagnosis of a pediatric stroke may prove to be challenging due to the relative rarity of the condition, lack of familiarity with pediatric stroke, and wide differential diagnoses.

## 2. Case Report

A five-year-old female presented to a pediatric emergency department (ED) after having two seizures. Earlier in the day the child had complained of a headache accompanied by vomiting. Ibuprofen offered minimal relief. During a nap later that day, the girl was noted to be having a tonic clonic seizure, lasting for an unknown duration. Emergency medical services (EMS) noted the patient to be postictal upon her arrival. While en route, EMS noted another seizure consisting of her right arm shaking and it was treated with lorazepam. Upon arrival to the ED, the patient again appeared to be postictal. There was no history of trauma prior to or during the seizure and no history of recent infectious illness. Her point of care blood glucose was normal. The patient had a history of seizures but had been seizure-free and off medication for two years. Her parents noted that this seizure was different than previous seizures in the fact that previous seizures were more localized.

The patient was born at full term and had no problems prenatally. Her past medical history was significant for seizure disorder and developmental delay. She had previously had a normal MRI of the brain and brain stem which showed no structural etiology for the existing seizure disorder or developmental delay. She was currently taking no medications. She was up to date on her immunizations and lived with her parents. Her family history was negative for seizure disorders.

On physical examination the child was lethargic. She followed commands, opened her eyes to voice, and answered questions with one word. Her initial vital signs were blood pressure 96/61 mm Hg, heart rate 85 beats/minute, respiratory rate 14 breaths/minute, temperature 96.8°F (tympanic), and oxygen saturation 98% on room air. Her head was normocephalic and atraumatic. Her pupils were equal, round, and reactive to light. Her neck was supple without meningeal signs. Her heart rate was regular rate and rhythm with no appreciable murmur. Her lungs were clear to auscultation bilaterally and her abdomen was soft, nontender, and nondistended. Her muscle strength appeared equal in all four extremities although she was not fully cooperative which was thought to be due to a postictal state. She had normal muscle tone and sensation to light touch in all four extremities. She had normal reflexes in all four extremities and no Babinski sign. No facial asymmetry was noted.

A computed tomography (CT) scan of the head without contrast was obtained as the patient complained of headache and her seizures were described as different from previous ones. CT scan showed loss of gray white matter differentiation in the right frontal lobe. A stat magnetic resonance imaging (MRI) was ordered as recommended by radiology; however this was delayed due to patient movement. The patient was admitted and scheduled for an MRI in the morning with sedation. MRI showed large areas of acute ischemia in the right anterior and middle cerebral artery distribution. A large, confluent area of restricted diffusions that involved the right frontal lobe, right medial temporal lobe, and the basal ganglia could be seen, making the diagnosis compatible with acute ischemic stroke. The patient was subsequently admitted to the pediatric intensive care unit. After a complicated clinical course she was discharged to a rehabilitation hospital at which time she was ambulatory with some residual focal weakness.

## 3. Discussion

To improve outcomes of pediatric stroke patients, emergency physicians need to increase awareness of incidence and diagnosis of pediatric stroke and its etiologies. It is estimated that 55% of strokes in children are ischemic and 45% are hemorrhagic [7]. Typical signs of anterior circulation stroke include unilateral weakness and/or sensory loss, loss of speech or language comprehension, and visual field deficit or focal seizures [5]. The relationship between seizures and stroke was documented as early as 1864 by John Hughlings Jackson; it has been more recently reported that seizures were 18 times more likely in children than adults within 24 hours of noted stroke symptoms [8, 9]. However, proportionately it is a rare occurrence; for instance, in our own institution, approximately 400 pediatric seizure patients were managed in the fiscal year prior to this isolated case of AIS. Obstacles to timely diagnosis of pediatric AIS include lack of experience with pediatric stroke in the ED, nonfocal presentation of stroke in children, wide differential diagnoses for childhood focal deficits, and poor sensitivity of acute CT scanning in the diagnosis of pediatric AIS [2]. Additionally, not all cases of pediatric seizures are scanned by a CT scan or MRI.

Treatment options are limited for the pediatric stroke patient. According to the American Heart Association (AHA) guidelines using tPA in children with acute ischemic stroke is a class III recommendation [7]. Due to the lack of clinical trials noting the risks and benefits of tPA in children, its use in the pediatric population is not supported at this time; however, its use could be considered in adolescents [7]. According to the AHA,

“…if the use of tPA is considered, then IV tPA should be administered within 3 hours and intra-arterial within six hours. The focus on secondary prevention of stroke is strongly emphasized in children since no approved acute treatments options are recommended. Treatment with low molecular weight heparin LMWH is preferred over unfractionated heparin (UFH). Starting treatment with the LMWH dose of 1 mg/kg given every twelve hours after the diagnosis of ischemic stroke with continuation for several weeks to months afterwards is recommending given the high likelihood of a hypercoagulability as a cause of ischemic stroke in children. Long-term treatment with warfarin is another alternative for secondary stroke prevention. Aspirin at a dose of 3–5 mg/kg per day is another option. Recommendations include treating hypoxia, hypoglycemia, anemia, fever, and hypotension. Prophylactic treatment of seizures is not recommended; however, therapeutic hypothermia is a class III recommendation [7].”

## 4. Why Should an Emergency Physician Be Aware of This?

Ultimately, combining physician awareness of pediatric AIS with inclusion of pediatric AIS in the differential diagnosis for pediatric patients presenting with seizures to the emergency department (especially when historically the type of seizure reported is different) may reduce the amount of time before a dedicated stroke team responds. With the emergency physician often being the first medical contact for a pediatric AIS patient, it is crucial to be aware of such cases and their management.
